# CDDO-Me Overcomes Gefitinib Resistance in NSCLC by Targeting the Src/STAT3 Axis to Induce Apoptosis and Pyroptosis

**DOI:** 10.3390/ijms27146481

**Published:** 2026-07-21

**Authors:** Tongtong Li, Weiyu Du, Ruoxian Wang, Xudong Yu, Bing Zhang, Wenjuan Wang, Jiahui Xu, Hui Cao, Dongtong Tang, Ning Liu

**Affiliations:** 1 International Research Centre for Food and Health, College of Food Science and Technology, Shanghai Ocean University, Shanghai 201306, China; prayer27t@163.com; 2 Department of Chemistry, College of Food Science and Technology, Shanghai Ocean University, Shanghai 201306, China; 18724091399@163.com (W.D.); 15226862577@163.com (R.W.); 19552770828@163.com (X.Y.); bling9803@163.com (B.Z.); 15225589092@163.com (W.W.); 18830396365@163.com (J.X.); ch2003030129@163.com (H.C.); tdtong0718@163.com (D.T.); 3 Marine Biomedical Science and Technology Innovation Platform of Lin-Gang Special Area, Shanghai 201306, China; 4 Shanghai Engineering Research Center of Aquatic-Product Processing & Preservation, Shanghai 201306, China

**Keywords:** non-small-cell lung cancer, CDDO-Me, Src/STAT3 pathway, gefitinib resistance, apoptosis, pyroptosis

## Abstract

Patients with *EGFR*-mutant non-small-cell lung cancer (NSCLC) develop acquired resistance to epidermal growth factor receptor tyrosine kinase inhibitor (EGFR-TKI), limiting the durability of targeted therapy. Bardoxolone methyl (CDDO-Me) has been reported to exert anti-inflammatory and anti-cancer activities. However, its role in acquired EGFR-TKI resistance remains unclear. Here, we found that CDDO-Me significantly enhanced sensitivity of resistant NSCLC cells to gefitinib, with combination index analysis confirming a synergistic interaction between CDDO-Me and gefitinib. Mechanistically, CDDO-Me induced mitochondrial dysfunction and reactive oxygen species (ROS) accumulation, thereby activating Caspase-3 mediated apoptosis and GSDME-dependent pyroptosis, as evidenced by increased lactate dehydrogenase (LDH) release. Network pharmacology and molecular docking analyses identified Src as a potential target of CDDO-Me. Cellular thermal shift assay (CETSA) confirmed cellular engagement between CDDO-Me and Src, and Western blot analysis showed that CDDO-Me suppressed Src/STAT3 signaling. Consistently, Src knockdown reduced the inhibitory effect of combined CDDO-Me and gefitinib treatment on colony formation and attenuated changes in apoptosis and pyroptosis regulatory proteins induced by the combination treatment. Collectively, these findings suggest that CDDO-Me enhances gefitinib sensitivity by targeting Src and suppressing Src/STAT3 signaling, leading to apoptosis and pyroptosis in gefitinib-resistant NSCLC cells. This study provides mechanistic evidence for further investigation of CDDO-Me-based combination strategies for gefitinib-resistant NSCLC.

## 1. Introduction

Non-small-cell lung cancer (NSCLC) is the most prevalent subtype of lung cancer, accounting for approximately 85% of all cases [1]. Targeted therapies, particularly epidermal growth factor receptor tyrosine kinase inhibitor (EGFR-TKI), have become a standard treatment option for advanced EGFR-mutant NSCLC [2,3,4,5]. First-generation EGFR-TKIs, including erlotinib and gefitinib, initially elicit substantial clinical responses and prolong progression-free survival [6]. However, the majority of patients ultimately develop acquired resistance, typically leading to disease relapse within 10–14 months after treatment initiation [7]. Such resistance limits the durability of targeted therapy in EGFR-mutant NSCLC and remains a major barrier to achieving a sustained therapeutic benefit [8,9]. Acquired resistance to EGFR-TKI in NSCLC is generally classified into EGFR-dependent and EGFR-independent mechanisms. EGFR-dependent resistance typically results from secondary alterations within EGFR, whereas EGFR-independent resistance is largely driven by the activation of alternative receptor tyrosine kinases, such as MET or HER2, and their downstream survival pathways, enabling tumor cells to escape EGFR blockade [10]. Although acquired resistance to EGFR-TKI can be initiated by diverse upstream events, it ultimately manifests as a reduced capacity for drug-induced tumor cell death. Among these associated alterations, diminished apoptotic sensitivity is particularly prevalent and remains pharmacologically tractable. Accordingly, targeting critical non-apoptotic pathways to restore apoptotic sensitivity may provide a therapeutic benefit. For instance, in ovarian cancer models, dual inhibition of EGFR and the JAK/STAT3 axis enhances apoptosis and increases sensitivity to EGFR-targeted therapy [11]. Similarly, evidence from prostate cancer models suggests that quercetin suppresses PI3K/AKT signaling while promoting apoptosis, thereby attenuating docetaxel-resistant phenotypes [12]. Pyroptosis, unlike apoptosis, has attracted increasing attention for its roles in inflammation and innate immunity [13]. Studies have shown that the activation of pyroptosis can trigger inflammatory responses that contribute to overcoming chemotherapeutic drug resistance [14]. Tanshinone I induces pyroptosis through the activation of canonical inflammatory signaling via the NF-κB/Caspase-3(8)/GSDME signaling pathway, thereby increasing cisplatin sensitivity [15]. Pyroptosis has garnered significant attention as a potential strategy for addressing drug resistance in cancer treatment [16]. In addition, studies have shown that cells with acquired resistance often evade the therapy-induced cell death through sustained activation of multiple pro-survival signaling pathways [17,18,19]. Therefore, identifying and targeting key targets within these persistently activated pro-survival signaling pathways are essential for elucidating resistance mechanisms and developing effective intervention strategies. Notably, Src is widely regarded as a central non-receptor tyrosine kinase that integrates diverse signaling networks associated with therapeutic resistance [20]. Src inhibition has been reported to reactivate pyroptotic cell death and reverse drug resistance in preclinical pancreatic cancer models [21]. However, the role of Src in mediating EGFR-TKI resistance in NSCLC remains poorly defined. Elucidating the functional roles and underlying mechanisms of Src in EGFR-TKI-resistant NSCLC may identify additional therapeutic targets and provide theoretical support for the development of combination treatment strategies.

Bardoxolone methyl (CDDO-Me), a synthetic oleanane triterpenoid, has been extensively investigated for its ability to regulate oxidative stress and inflammatory signaling pathways [22]. The binding of CDDO-Me to IKKβ inhibits NF-κB activation and downstream proinflammatory signaling pathways [23]. CDDO-Me has demonstrated anti-tumor activity in preclinical models across multiple cancer types. However, its therapeutic potential in EGFR-TKI-resistant NSCLC cells requires further study. Herein, we found that CDDO-Me combined with gefitinib effectively inhibited the proliferation of gefitinib-resistant NSCLC cells both in vitro and in vivo. Mechanistically, the combination of CDDO-Me and gefitinib inhibited Src/STAT3 signaling, leading to increased mitochondrial ROS generation and the subsequent induction of apoptosis and GSDME-mediated pyroptosis, thereby reversing gefitinib resistance. These findings suggest that combined treatment with CDDO-Me and gefitinib may represent a potential strategy to improve therapeutic responses in gefitinib-resistant NSCLC.

## 2. Results

### 2.1. CDDO-Me Restores Gefitinib Sensitivity in Gefitinib-Resistant NSCLC Cells In Vitro

**Figure 1:**
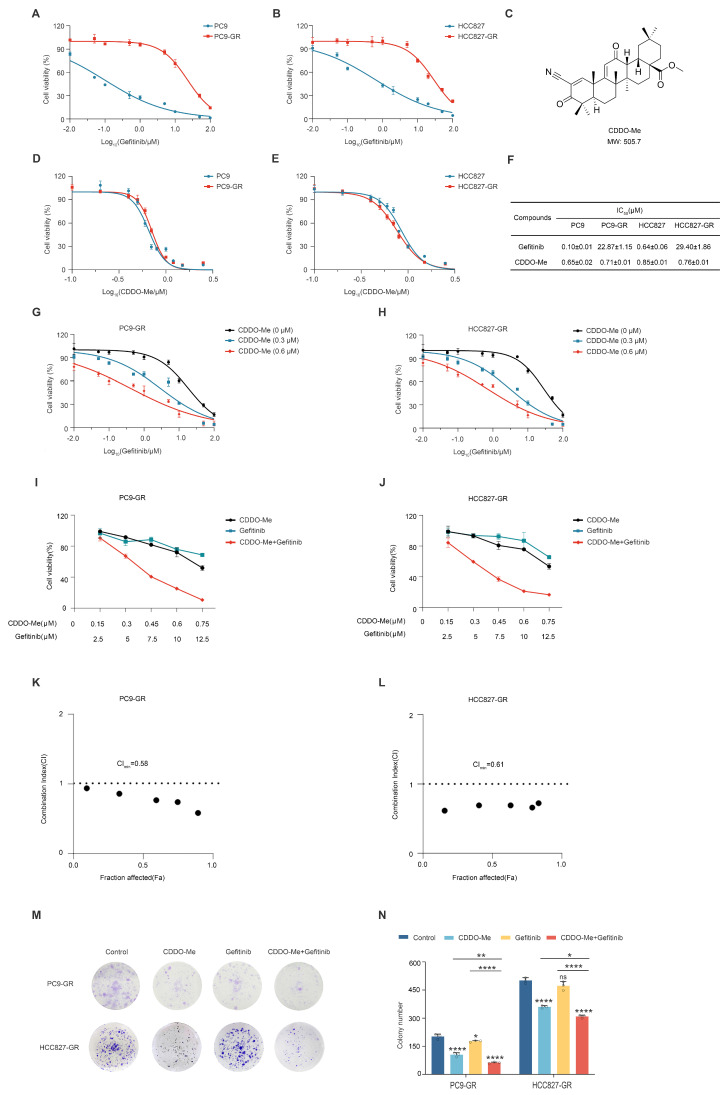
CDDO-Me overcomes gefitinib resistance in NSCLC cells in vitro. (**A**,**B**) Dose–response curves for gefitinib in parental PC9 and HCC827 cells and their gefitinib-resistant cell lines (PC9-GR, HCC827-GR). Cells were treated with increasing concentrations of gefitinib for 72 h, and cell viability was measured by CCK-8 assay. (**C**) Chemical structure of CDDO-Me. (**D**,**E**) Dose–response curves for CDDO-Me in parental and gefitinib-resistant NSCLC cells treated for 48 h and analyzed by CCK-8 assay. (**F**) IC_50_ values of gefitinib and CDDO-Me in parental and resistant cell lines calculated from the curves in (**A**,**B**,**D**,**E**). (**G**,**H**) Effects of combined treatment with CDDO-Me and gefitinib on cell viability in PC9-GR and HCC827-GR cells. Cells were incubated with increasing concentrations of gefitinib in the presence of the indicated fixed concentrations of CDDO-Me for 48 h, and cell viability was determined by CCK-8 assay. (**I**,**J**) Cell viability of PC9-GR and HCC827-GR cells treated with CDDO-Me, gefitinib, or their combination at the indicated fixed-ratio concentrations for 48 h. Cell viability was determined using the CCK-8 assay. (**K**,**L**) Combination index (CI) plots for CDDO-Me and gefitinib in PC9-GR and HCC827-GR cells, calculated using CompuSyn software based on the Chou–Talalay method. The dotted horizontal line indicates CI = 1. CI values below 1 indicate synergistic interactions between CDDO-Me and gefitinib. (**M**,**N**) Colony formation of PC9-GR and HCC827-GR cells treated with vehicle control, CDDO-Me (0.6 μM), gefitinib (10 μM), or the combination of CDDO-Me (0.6 μM) and gefitinib (10 μM) for 10–14 days; representative colony images (**M**) and quantitative analysis of colony numbers (**N**) are shown. All data are shown as mean ± SD and were analyzed by two-way ANOVA followed by Tukey’s multiple-comparison test. * *p* < 0.05, ** *p* < 0.01, **** *p* < 0.0001.

We established acquired gefitinib-resistant models in vitro by continuously exposing parental PC9 and HCC827 cells to stepwise-escalating concentrations of gefitinib. Gefitinib sensitivity was assessed using CCK-8 assays. Compared with the parental cell lines, the resistant cell lines displayed markedly reduced responsiveness, with IC_50_ values increased by approximately 200-fold in PC9-GR cells and 45-fold in HCC827-GR cells, confirming successful establishment of the resistant cells (Figure 1A,B,F). In parallel, we assessed the effect of CDDO-Me (structure shown in Figure 1C) on the viability of parental and gefitinib-resistant NSCLC cells. As shown in Figure 1D,E,F, CDDO-Me significantly inhibited the viability of all tested cell lines, including both parental and gefitinib-resistant cells. The comparable IC_50_ values between parental and resistant cells further indicated that gefitinib-resistant cells did not exhibit apparent cross-resistance to CDDO-Me. Furthermore, in the presence of CDDO-Me, the gefitinib IC_50_ in PC9-GR cells decreased from 18.720 μM to 3.020 μM and 0.433 μM, and the corresponding values in HCC827-GR cells decreased from 29.200 μM to 3.160 μM and 0.724 μM. Treatment with 0.6 μM CDDO-Me reduced the IC_50_ of gefitinib by approximately 43-fold in PC9-GR cells and 40-fold in HCC827-GR cells, suggesting that CDDO-Me markedly enhanced gefitinib sensitivity in resistant NSCLC cells (Figure 1G,H). To further evaluate the interaction between CDDO-Me and gefitinib, combination index analysis was performed using CompuSyn software (version 1.2). The CI values were below 1 at the tested effective concentrations, supporting a synergistic interaction between CDDO-Me and gefitinib in gefitinib-resistant NSCLC cells (Figure 1I–L). Colony formation assays further confirmed that CDDO-Me alone significantly impaired the colony formation abilities of PC9-GR and HCC827-GR cells, whereas co-treatment with CDDO-Me and gefitinib resulted in the most pronounced suppression of colony formation, consistent with the synergistic inhibitory effect indicated by the CI analysis (Figure 1M,N). Additionally, wound-healing assays revealed that gefitinib monotherapy maintained a relatively high wound-closure rate, indicating preserved migratory capacity in both PC9-GR and HCC827-GR cells. In contrast, CDDO-Me treatment markedly impaired cell migration. Notably, combined CDDO-Me and gefitinib treatment further reduced migratory activity compared with either monotherapy, suggesting a potential role for this combination in attenuating the invasive potential of gefitinib-resistant NSCLC cells (Figure S1A,B). These findings demonstrate that CDDO-Me enhances the sensitivity of NSCLC cells to gefitinib, thereby providing a potential therapeutic strategy for the treatment of NSCLC.

### 2.2. CDDO-Me in Combination with Gefitinib Significantly Suppresses Tumor Growth of Gefitinib-Resistant Xenografts In Vivo

**Figure 2:**
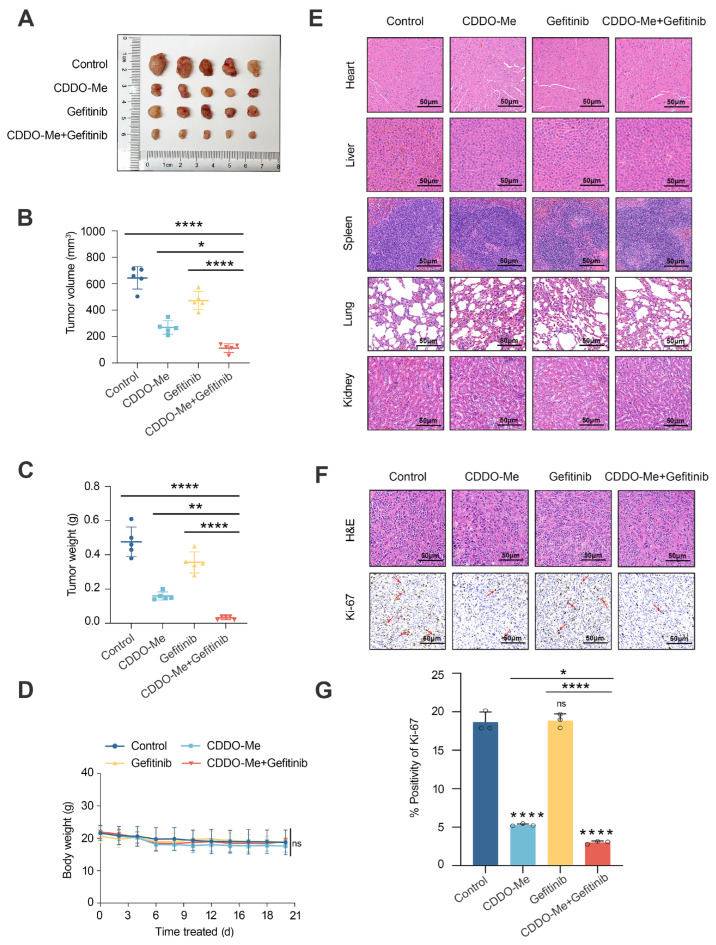
CDDO-Me overcomes gefitinib resistance in NSCLC in vivo. Nude mice bearing PC9-GR xenografts (n = 5 per group) were randomly assigned to four treatment groups and treated once every two days for 21 days with intraperitoneal injections of control (DMSO), CDDO-Me (7 mg/kg), gefitinib (10 mg/kg), or the combination of CDDO-Me (7 mg/kg) and gefitinib (10 mg/kg). (**A**) Images of tumors from each group at the end of treatment. (**B**) Tumor volume of xenografts in each group during the treatment period. (**C**) Final tumor weight of xenografts in each group at the end of treatment. (**D**) Body weight of mice in each treatment group during the 21-day treatment. (**E**) H&E staining of major organs (heart, liver, spleen, lung, kidney) collected from mice after treatment (magnification × 400, scale bar: 50 μm). (**F**) H&E staining and Ki-67 immunohistochemical staining of tumor sections from each group (magnification × 400, scale bar: 50 μm). (**G**) Quantitative analysis of Ki-67-positive cells. All data are shown as mean ± SD and were analyzed by one-way ANOVA followed by Tukey’s multiple-comparison test. ns, not significant, * *p* < 0.05, ** *p* < 0.01, **** *p* < 0.0001.

To determine whether CDDO-Me can overcome gefitinib resistance in vivo, we established a xenograft model by subcutaneously implanting gefitinib-resistant PC9-GR cells into the flanks of BALB/c nude mice. When the tumor volume reached approximately 100 mm^3^, the mice were randomized into four groups and treated intraperitoneally with DMSO, CDDO-Me (7 mg/kg), gefitinib (10 mg/kg), or a combination of CDDO-Me and gefitinib once every two days for 21 days (Figure 2A). Tumor volume and body weight were monitored every 2 days. Combination treatment with CDDO-Me and gefitinib markedly decreased tumor volume and weight compared with the control and monotherapy groups, supporting the efficacy of CDDO-Me in suppressing the growth of gefitinib-resistant NSCLC tumors (Figure 2B,C). During the 21-day treatment period, body weight remained comparable among mice in the control, gefitinib, CDDO-Me, and combination treatment groups (Figure 2D). H&E staining further revealed no obvious histopathological abnormalities in major organs from mice in these groups (Figure 2E). These observations provide preliminary evidence that the combination treatment was well tolerated in this xenograft model. Furthermore, immunohistochemical analysis showed that Ki-67 expression was decreased in the CDDO-Me monotherapy group and combination treatment group relative to the control and gefitinib monotherapy groups, with the greatest reduction observed following combination therapy (Figure 2F,G). These in vivo findings were consistent with our in vitro observations and indicate that CDDO-Me enhances the anti-tumor efficacy of gefitinib in resistant NSCLC, thereby supporting its potential utility in overcoming EGFR-TKI resistance.

### 2.3. CDDO-Me Enhances Sensitivity to Gefitinib by Inducing Apoptosis and Pyroptosis in PC9-GR and HCC827-GR Cells

**Figure 3:**
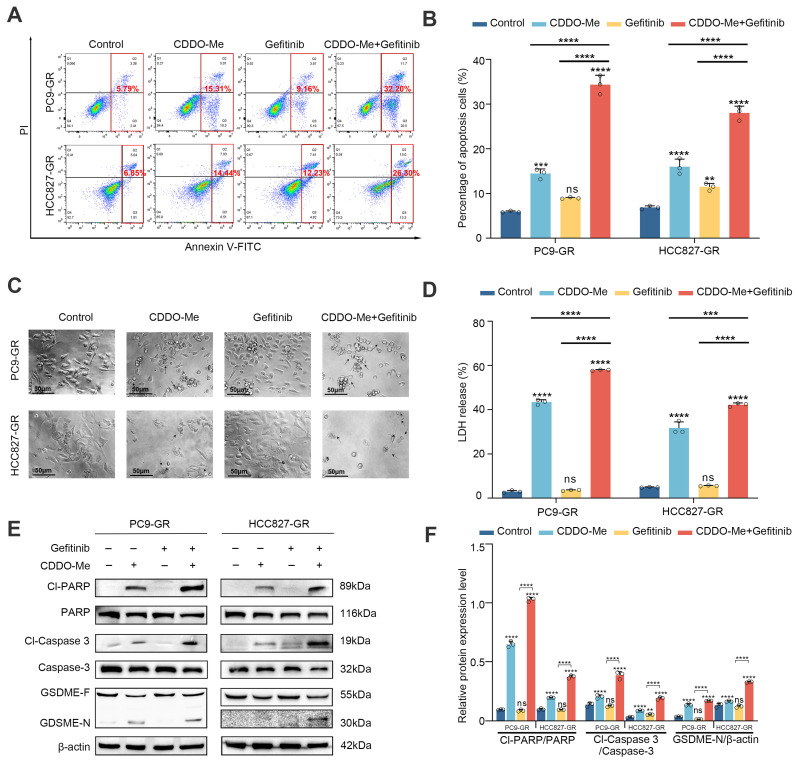
CDDO-Me induces apoptosis and pyroptosis in gefitinib-resistant NSCLC cells by triggering cleavage of Caspase-3 and GSDME. (**A**) PC9-GR and HCC827-GR cells were treated with CDDO-Me (0.6 μM) and gefitinib (10 μM), alone or in combination, for 24 h, and apoptosis was measured by Annexin V-FITC/PI double staining and flow cytometry. (**B**) Quantitative analysis of apoptotic cells in PC9-GR and HCC827-GR cells treated as in (**A**). (**C**) Representative bright-field images of PC9-GR and HCC827-GR cells treated as in (**A**), showing cell morphology under the different treatment conditions (scale bar, 50 μm). The black arrowheads indicated the characteristic balloons on the cell membrane. (**D**) LDH release in the culture supernatant of PC9-GR and HCC827-GR cells treated as in (**A**) was measured by LDH assay, and the results are expressed as a percentage of total LDH. (**E**) Western blot analysis of Cl-PARP, PARP, Cl-Caspase 3, Caspase-3, GSDME-F and GSDME-N in PC9-GR and HCC827-GR cells treated as in (**A**). (**F**) Quantitative analysis of Cl-PARP/PARP, Cl-Caspase 3/Caspase-3 and GSDME-N/β-actin protein levels shown in (**E**). All data are shown as mean ± SD, two-way ANOVA, ns, not significant, ** *p* < 0.01, *** *p* < 0.001, **** *p* < 0.0001.

Given the critical role of re-inducing cell death in anti-tumor responses and the reversal of drug resistance, we next investigated whether CDDO-Me restores sensitivity to cell death in gefitinib-resistant NSCLC cells by promoting apoptosis and GSDME-dependent pyroptosis. Flow cytometry was employed to evaluate the apoptotic rate of NSCLC cells following treatment with CDDO-Me, gefitinib, or their combination. After 24 h of treatment, CDDO-Me markedly increased apoptosis in both gefitinib-resistant NSCLC cell lines. Specifically, CDDO-Me increased the apoptotic rate from 5.79% to 15.31% in PC9-GR cells and from 6.85% to 14.44% in HCC827-GR cells. Gefitinib monotherapy induced a relatively modest increase in apoptosis, reaching 9.16% in PC9-GR cells and 12.23% in HCC827-GR cells. Importantly, combined treatment with CDDO-Me and gefitinib further elevated apoptosis to 32.20% in PC9-GR cells and 26.30% in HCC827-GR cells, indicating that CDDO-Me enhanced gefitinib-induced apoptosis in gefitinib-resistant NSCLC cells (Figure 3A,B). Our findings indicate that co-treatment with CDDO-Me and gefitinib can effectively induce apoptosis in gefitinib-resistant NSCLC cells. Previous studies have demonstrated that apoptosis and pyroptosis are mechanistically interconnected [24]. Cl-Caspase 3, a key executor of apoptosis, has been demonstrated to initiate pyroptotic cell death by cleaving and activating GSDME, thereby converting apoptosis into secondary pyroptosis [25,26]. Consequently, we examined whether the enhanced apoptotic response induced by CDDO-Me was accompanied by pyroptotic morphological changes. Bright-field microscopy revealed that cells treated with CDDO-Me alone or in combination with gefitinib exhibited characteristic pyroptotic morphology, including plasma membrane rupture, cytoplasmic swelling, and bubble-like protrusions (Figure 3C). In contrast, the control and gefitinib groups maintained intact cellular morphology with smooth plasma membranes. To further quantify membrane integrity loss, LDH release assays were performed. Extracellular LDH activity is commonly used as a readout of membrane disruption and lytic cell death and is consistent with pyroptotic membrane rupture. Consistent with the observed morphological changes, LDH levels were significantly elevated in both the CDDO-Me monotherapy and combination groups compared with the control and gefitinib monotherapy groups. Moreover, LDH release was significantly higher following combination treatment than following CDDO-Me monotherapy, indicating a more apparent pyroptotic response (Figure 3D). Based on these results, we examined the expression of key proteins involved in the regulation of apoptotic and pyroptotic pathways underlying CDDO-Me-mediated reversal of gefitinib resistance. Our results revealed that the protein expression levels of cleaved PARP and cleaved Caspase-3 were increased following CDDO-Me treatment alone or in combination with gefitinib, consistent with the activation of apoptosis. Meanwhile, combined CDDO-Me and gefitinib treatment downregulated the expression of full-length GSDME protein (GSDME-F) and upregulated the expression of the GSDME N-terminal fragment (GSDME-N) compared with the control and gefitinib monotherapy groups (Figure 3E,F). This finding suggests that CDDO-Me induces pyroptosis in gefitinib-resistant NSCLC cells through GSDME cleavage. In conclusion, our results suggest that the combination of CDDO-Me and gefitinib treatment enhances drug responsiveness in resistant NSCLC cells by promoting apoptosis and pyroptosis.

### 2.4. CDDO-Me Induces Mitochondrial Dysfunction and ROS Generation

**Figure 4:**
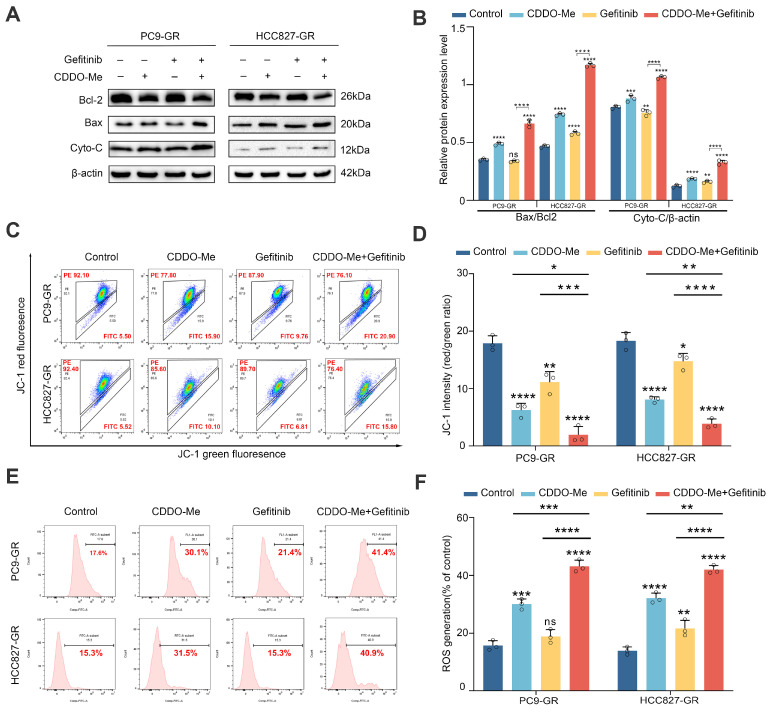
CDDO-Me induces mitochondrial dysfunction and ROS accumulation in gefitinib-resistant NSCLC cells. (**A**) PC9-GR and HCC827-GR cells were treated with CDDO-Me (0.6 μM) and gefitinib (10 μM), alone or in combination, for 24 h, and the expression of mitochondrial apoptosis-related proteins (Bcl-2, Bax, and Cyto-C) was examined by Western blotting. (**B**) Quantitative analysis of Bcl-2, Bax and Cyto-C expression levels in PC9-GR and HCC827-GR cells shown in (**A**). (**C**) MMP was assessed by JC-1 staining followed by flow cytometric analysis in PC9-GR and HCC827-GR cells treated as in (**A**). (**D**) Quantitative analysis of the JC-1 red/green fluorescence ratio in PC9-GR and HCC827-GR cells treated as in (**A**). (**E**) Intracellular ROS levels were measured by flow cytometry using the DCFH-DA probe in PC9-GR and HCC827-GR cells treated as in (**A**); representative histograms are shown. (**F**) Quantitative analysis of intracellular ROS levels in PC9-GR and HCC827-GR cells shown in (**E**), expressed as a percentage of the control. Data are presented as mean ± SD from three independent experiments, two-way ANOVA, ns, not significant, * *p* < 0.05, ** *p* < 0.01, *** *p* < 0.001, **** *p* < 0.0001.

Mitochondria are closely associated with programmed cell death, including classical apoptosis and GSDME-dependent pyroptosis. To elucidate how CDDO-Me induces cell death in gefitinib-resistant NSCLC cells, we examined whether the mitochondrial apoptotic pathway was activated by assessing the expression of Bcl-2, Bax, and Cyto-C. Western blot analysis demonstrated that CDDO-Me decreased the expression of the apoptosis suppressor Bcl-2 while increasing the levels of the apoptosis promoting proteins Bax and Cyto-C, with the most robust effects observed following combined treatment with gefitinib (Figure 4A,B). These changes in mitochondrial apoptosis related proteins suggested the involvement of mitochondrial apoptotic signaling. To further assess mitochondrial dysfunction, the effect of CDDO-Me on mitochondrial membrane potential (MMP) was examined using JC-1 staining. Compared with the control and gefitinib monotherapy groups, cells treated with CDDO-Me alone or in combination with gefitinib exhibited a significant reduction in the JC-1 red/green fluorescence ratio, consistent with enhanced mitochondrial depolarization (Figure 4C,D). Given that MMP is tightly linked to ROS accumulation, and that ROS have been implicated in both apoptosis and GSDME-dependent pyroptosis, we further assessed whether ROS are involved in the cell death triggered by CDDO-Me in gefitinib-resistant NSCLC cells. Flow cytometric analysis using the DCFH-DA probe confirmed that intracellular ROS levels were increased in gefitinib-resistant NSCLC cells following CDDO-Me treatment and were further elevated by co-treatment with gefitinib (Figure 4E,F). In summary, CDDO-Me induces mitochondrial depolarization and enhances intracellular ROS generation, thereby activating the Caspase-3/GSDME pathway and promoting mitochondria-dependent apoptotic and pyroptotic cell death in gefitinib-resistant NSCLC cells.

### 2.5. Src as a Potential Target for Overcoming Gefitinib Resistance in NSCLC

**Figure 5:**
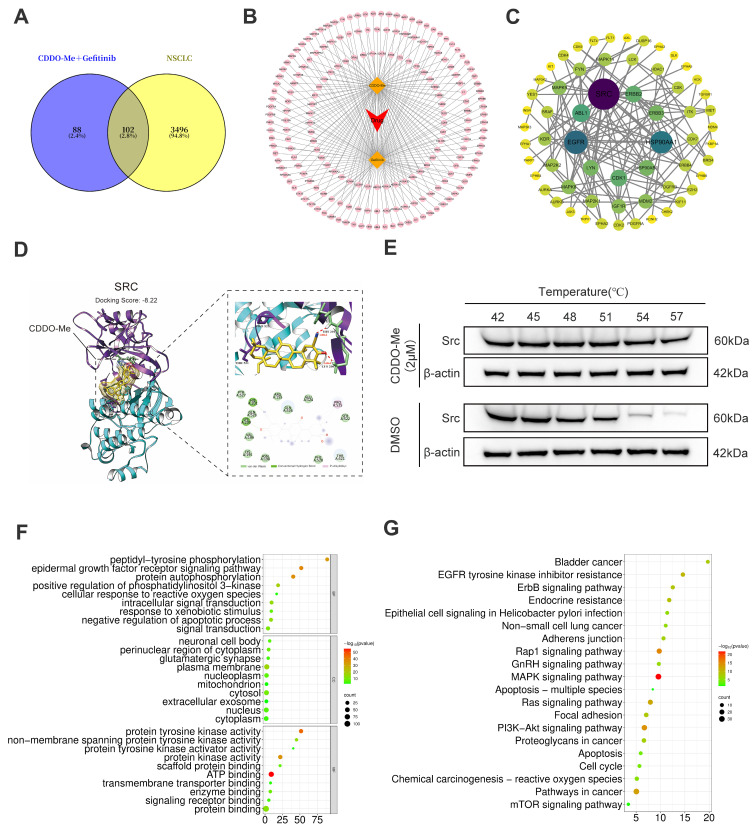
Integrated network pharmacology and molecular docking analyses identify Src as a key target of CDDO-Me in gefitinib-resistant NSCLC. (**A**) Venn diagram showing the overlap between the predicted targets of the combination of CDDO-Me and gefitinib and NSCLC-associated genes. (**B**) Construction of the drug-target network. The central nodes represent CDDO-Me and gefitinib, and the surrounding circles represent their predicted protein targets. (**C**) PPI network of the 102 overlapping targets constructed using STRING, highlighting Src as a central hub node. (**D**) Predicted binding mode of CDDO-Me with Src obtained by molecular docking. Left: three-dimensional view of CDDO-Me in the Src active pocket; right: enlarged view and 2D interaction diagram showing hydrogen bonds and van der Waals contacts with key residues, together with the docking score. (**E**) CETSA analysis of Src thermal stability in PC9-GR cells treated with DMSO or CDDO-Me (2 μM). Cell lysates were heated at the indicated temperatures ranging from 42 to 57 °C, and Src protein levels after heat treatment were detected by Western blotting. (**F**) Gene Ontology (GO) enrichment analysis of the 102 overlapping targets. Significantly enriched GO terms in the biological process, cellular component and molecular function categories are shown. (**G**) KEGG pathway enrichment analysis of the 102 overlapping targets. Significantly enriched pathways related to apoptosis and cancer-associated signaling are displayed.

Based on the preceding results demonstrating that the combination of CDDO-Me and gefitinib induces apoptosis and pyroptosis via mitochondrial dysfunction, we next sought to identify the key upstream signaling targets underlying this effect. Using SwissTargetPrediction, 190 predicted compound-associated targets were obtained for CDDO-Me and gefitinib (Supplementary Table S1). Meanwhile, 3598 NSCLC related genes were collected from disease associated databases. As illustrated in Figure 5A, 102 overlapping targets were identified by integrating the 190 predicted compound associated targets and 3598 NSCLC-related genes, which were considered potential targets of the combined treatment (Supplementary Table S2). A drug-target interaction network was subsequently constructed and visualized using Cytoscape (version 3.10.3). The overlapping targets were further imported into the STRING database to analyze physical and functional protein-protein interaction (PPI) networks. This analysis revealed that Src occupied a central hub position within the network (Figure 5B,C), suggesting a pivotal role in the mechanism by which CDDO-Me overcomes gefitinib resistance. To further assess the potential direct interaction between Src and CDDO-Me, molecular docking analyses were performed to evaluate binding modes and identify key interacting residues (Figure 5D). The binding model generated by molecular docking showed that two hydrogen bonds were formed between CDDO-Me and the residues His201 and Lys200, which are located within the Src active site. AutoDock (version 1.2.4)-based molecular docking showed that CDDO-Me bound to Src with a docking score of −8.22 kcal/mol, indicating a favorable interaction between CDDO-Me and Src. Meanwhile, multiple van der Waals interactions were also observed within the active site between CDDO-Me and residues including PTR527, ARG155, GLU524, GLN526, GLN528, VAL199, LYS195, ASN198, PHE520, THR521, and SER522, which may contribute to the stabilization of its binding conformation and enhanced binding stability. Given that ligand binding can alter the thermal stability of target proteins, we next performed CETSA to assess cellular target engagement. Temperature-dependent immunoblotting showed that CDDO-Me increased the thermal stability of Src compared with the DMSO control (Figure 5E). Together, the CETSA results provide cellular evidence for the interaction between CDDO-Me and Src, further supporting the molecular docking results. Gene Ontology (GO) enrichment analysis of the 102 potential targets was then performed, and the results were visualized using an online bioinformatics platform (Figure 5F). GO enrichment analysis of the 102 overlapping targets showed that the enriched terms covered multiple biological processes, cellular components, and molecular functions, particularly functions related to signaling pathways and kinase activity. Apoptosis-related regulation was also represented among the enriched biological processes. KEGG pathway enrichment analysis showed that the overlapping targets were enriched in a broad range of cancer associated and drug-resistance related pathways, including pathways involved in EGFR-TKI resistance, kinase mediated signaling, cell cycle regulation, and apoptosis (Figure 5G). Further analysis of the KEGG pathway gene membership showed that Src was involved in several enriched pathways associated with cancer and drug resistance, including EGFR tyrosine kinase inhibitor resistance, endocrine resistance, the ErbB signaling pathway, focal adhesion, proteoglycans in cancer, and chemical carcinogenesis related to reactive oxygen species (Supplementary Table S3). This further supports the potential relevance of Src in the network pharmacology results. Previous studies have shown that Src acts as an upstream negative regulator of drug-induced pyroptosis, and that pharmacological inhibition of Src has been reported to reactivate pyroptosis and overcome chemoresistance in cancer. Collectively, these findings suggest that Src may play a critical role in mediating the cell death response to CDDO-Me, contributing to the induction of both apoptosis and pyroptosis.

### 2.6. CDDO-Me Enhances NSCLC Sensitivity to Gefitinib by Suppressing the Src/STAT3 Signaling Pathway In Vitro and In Vivo

**Figure 6:**
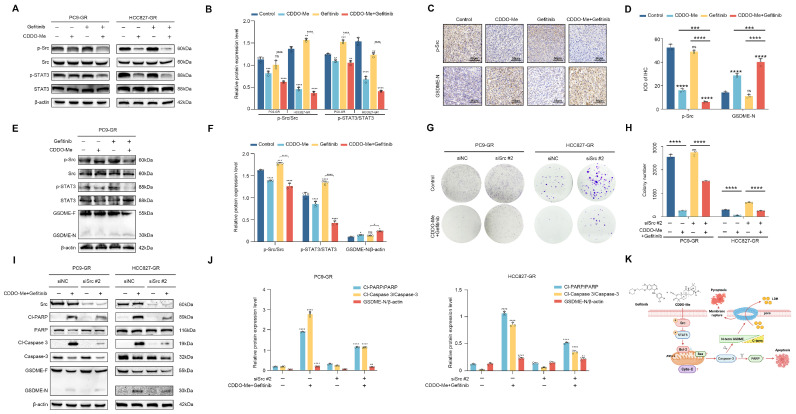
The Src/STAT3 signaling pathway is involved in CDDO-Me overcoming gefitinib resistance in vitro and in vivo. (**A**) PC9-GR and HCC827-GR cells were treated with gefitinib and CDDO-Me, alone or in combination, for 24 h as indicated, and the phosphorylation of Src and STAT3 was examined by Western blotting. (**B**) Quantitative analysis of p-Src/Src and p-STAT3/STAT3 expression levels in PC9-GR and HCC827-GR cells. (**C**) Representative immunohistochemical staining of tumor tissues from nude mice for p-Src and GSDME in the control, CDDO-Me, gefitinib and combination groups (magnification, 400×; scale bar, 50 μm). (**D**) Quantitative measurement of p-Src and GSDME immunohistochemical staining was performed using Image-Pro Plus 6.0 (n = 5 fields of view). (**E**) Western blot analysis of p-Src, Src, p-STAT3, STAT3, GSDME-F and GSDME-N in tumor tissues. (**F**) Quantitative measurement of p-Src/Src, p-STAT3/STAT3 and GSDME-N/β-actin expression levels in tumor tissues. (**G**,**H**) PC9-GR and HCC827-GR cells were transfected with control siRNA (siNC) or Src-targeting siRNA (siSrc #2). After 24 h, cells were cultured in control medium or in the presence of CDDO-Me combined with gefitinib for 10–14 days. Representative images of crystal violet-stained colonies are shown in (**G**), and colony numbers are quantified in (**H**). (**I**) PC9-GR and HCC827-GR cells were transfected with siNC or siSrc #2 and then treated with CDDO-Me combined with gefitinib for 24 h. The expression of Src, Cl-PARP, PARP, Cl-Caspase 3, Caspase-3 and GSDME-N was evaluated by Western blotting. (**J**) Quantitative analysis of Cl-PARP/PARP, Cl-Caspase 3/Caspase-3 and GSDME-N ratios in (**I**). (**K**) Graphical model for the molecular mechanism by which CDDO-Me overcomes gefitinib resistance in NSCLC cells. The T-shaped arrows represent inhibition, while the pointed arrows indicate activation or stimulation. All data are shown as mean ± SD, two-way ANOVA (for grouped data), ns, not significant, * *p* < 0.05, ** *p* < 0.01, *** *p* < 0.001, **** *p* < 0.0001.

Based on the target screening results derived from network pharmacology and molecular docking analyses, we further validated Src as a functional target of CDDO-Me by examining whether CDDO-Me inhibits Src activation and its downstream signaling, thereby sensitizing gefitinib-resistant NSCLC cells to gefitinib. In both PC9-GR and HCC827-GR cells, CDDO-Me alone or in combination with gefitinib significantly suppressed the phosphorylation levels of Src and STAT3 compared with the control and gefitinib monotherapy groups (Figure 6A,B). These observations were subsequently extended in vivo. Immunohistochemical analysis of gefitinib-resistant xenograft tumors demonstrated that CDDO-Me treatment decreased p-Src staining and increased GSDME-N positivity, and these changes were further enhanced when CDDO-Me was combined with gefitinib (Figure 6C,D). Consistently, Western blot analysis of tumor tissues confirmed that co-treatment with CDDO-Me and gefitinib resulted in downregulation of p-Src and p-STAT3, accompanied by upregulation of GSDME-N (Figure 6E,F). Together, these in vitro and in vivo findings indicate that CDDO-Me inhibits the Src/STAT3 signaling pathway, thereby promoting apoptosis and GSDME-dependent pyroptosis and contributing to the reversal of gefitinib resistance. To further determine whether Src was required for the sensitizing effect of CDDO-Me, Src knockdown was performed in PC9-GR and HCC827-GR cells (Figure S2). In siNC cells, colony formation was markedly suppressed by combined CDDO-Me and gefitinib treatment (Figure 6G,H), accompanied by increased levels of cleaved PARP, cleaved Caspase-3, and GSDME-N (Figure 6I,J). However, following Src knockdown, the inhibitory effect of the combination treatment on colony formation was partially attenuated compared with that in siNC cells. Similarly, the increases in cleaved PARP, cleaved Caspase-3, and GSDME-N induced by the combination treatment were reduced after Src knockdown. These results indicate that Src contributes to the enhanced response of gefitinib-resistant NSCLC cells to combined CDDO-Me and gefitinib treatment. Collectively, these findings suggest that CDDO-Me overcomes gefitinib resistance by inducing apoptosis and GSDME-mediated pyroptosis, at least partly through inhibition of the Src/STAT3 signaling pathway (Figure 6K).

## 3. Discussion

EGFR-TKIs have markedly improved the outcomes of patients with EGFR-mutant NSCLC. However, nearly all patients ultimately develop acquired resistance, which remains a major obstacle to further gains in survival [27,28]. In this study, we demonstrated that CDDO-Me in combination with gefitinib markedly enhanced anti-tumor activity in gefitinib-resistant NSCLC, resulting from activation of apoptosis and induction of GSDME-mediated pyroptosis.

To date, among the approaches used to overcome acquired EGFR-TKI resistance, combination therapies have become a major focus of investigation [29]. Combinations of EGFR-TKI with monoclonal antibodies, chemotherapy and other TKIs have been reported to overcome resistance to EGFR-TKI [30]. Multiple studies have shown that combining EGFR-TKI with small-molecule inhibitors targeting PI3K/AKT, ERK, or NF-κB signaling pathways can restore EGFR-TKI sensitivity in EGFR-mutant NSCLC [12,31]. In contrast to prior combination strategies that primarily seek to restore sensitivity to EGFR-TKIs by additively suppressing proliferative and survival signaling, our study emphasizes reshaping how resistant cells execute death, with particular attention to lytic cell death associated with pyroptotic features as a mechanism for restoring drug sensitivity. Cell death is increasingly recognized as a complex regulatory network and can be broadly classified into programmed cell death and inflammatory cell death [32]. Although apoptosis has been extensively investigated in drug-resistant cells, cell death may transition from a relatively apoptotic process to secondary pyroptosis, highlighting pyroptosis as a mechanistically relevant mode of execution for understanding and overcoming drug resistance [33,34]. Gasdermin (GSDM) family proteins (including GSDMs A–E) are key effectors that mediate pyroptosis [35]. GSDMB displays a tissue-selective expression pattern, with prominent expression in gastrointestinal epithelial cells and tumors of gastrointestinal origin [36]. By contrast, GSDMD shows a broader expression distribution across cancer types, making it a commonly used mechanistic axis for pyroptosis-based therapy [37]. Notably, GSDME has been reported to be widely expressed across molecular subtypes of lung cancer [38]. In NSCLC, GSDME-mediated pyroptosis has been shown to enhance cisplatin sensitivity and contribute to tumor regression, accompanied by increased anti-tumor immune cell infiltration [39]. In this study, we found that, compared with the control and either monotherapy, combined treatment with CDDO-Me and gefitinib induced Caspase-3 cleavage, which in turn mediated GSDME cleavage and the induction of pyroptosis. In contrast, the protein levels of GSDMB and GSDMD remained unchanged. These findings indicate that pyroptosis induced by combined CDDO-Me and gefitinib treatment in gefitinib-resistant NSCLC cells is predominantly mediated by GSDME. Given that GSDME-mediated pyroptosis is associated with the endogenous mitochondrial apoptotic pathway, MMP was assessed by JC-1 staining, and a marked loss of MMP was observed in the groups treated with the combination of CDDO-Me and gefitinib. MMP loss is closely linked to ROS accumulation. Therefore, mitochondrial ROS were further evaluated using a mitochondrial probe, and elevated ROS levels were detected in the combination treatment group. Our results demonstrated that the combination treatment induced mitochondrial dysfunction and activated the apoptotic pathway. Taken together, our data indicate that CDDO-Me overcomes EGFR-TKI resistance by triggering apoptosis and subsequent GSDME-mediated secondary pyroptosis.

Given that the development of cancer results from the combined action of multiple factors, multi-target small molecules may offer potential advantages in combination therapy and the reversal of drug resistance [40]. CDDO-Me is a multifunctional small molecule that modulates multiple signaling pathways, including activation of the Keap1/Nrf2 axis and inhibition of NF-κB signaling, thereby impacting tumor growth and survival [22,41,42]. In this study, we used network pharmacology to systematically screen the potential novel targets of CDDO-Me and gefitinib, integrated them with NSCLC disease targets to construct a PPI network, and identified Src as a potential key target for therapeutic strategies for NSCLC. Molecular docking also indicated a good binding affinity between CDDO-Me and Src. Western blot showed that the combination treatment of CDDO-Me and gefitinib suppressed the phosphorylation of Src and STAT3. Moreover, Src knockdown significantly attenuated the inhibitory effect of combined CDDO-Me and gefitinib treatment on colony formation. Consistently, the upregulation of Cleaved PARP, Cleaved Caspase-3, and GSDME-N protein levels induced by CDDO-Me alone or in combination with gefitinib was significantly attenuated by Src knockdown. These results further confirm that Src is required for the ability of CDDO-Me to overcome gefitinib resistance in NSCLC. Nevertheless, the PPI network also highlighted other candidate targets such as HSP90, ErbB2 and ErbB3, suggesting that they may be involved in EGFR-TKI resistance in NSCLC. However, their relevance as key targets for overcoming resistance requires further validation.

## 4. Materials and Methods

### 4.1. Cell Lines and Cell Culture

The PC9 (ATCC, CRL-32727, Manassas, VA, USA) and HCC827 (ATCC, CRL-2868, Manassas, VA, USA) cell lines used in this study were obtained from the American Type Culture Collection. Both cell lines were cultured in RPMI-1640 medium (#350-000-CL, WISENT CORPORATION, Nanjing, China) supplemented with 10% fetal bovine serum (FBS, Gibco, Carlsbad, CA, USA) and 1% penicillin–streptomycin (P/S) dual antibiotics (15140-122, Gibco, Carlsbad, CA, USA) and maintained at 37 °C in a 5% CO_2_ incubator. All cell lines were routinely tested to confirm that they were free of Mycoplasma. All cell-handling procedures were conducted in a biosafety cabinet under strict aseptic conditions. Prior to experimentation, cells in the logarithmic growth phase and in optimal condition were selected for subsequent experiments. To generate gefitinib-resistant PC9 and HCC827 sublines, the cells were initially treated with 0.01 µM gefitinib (CAS 184475-35-2, 99.94% purity, #HY-50895, MedChemExpress LLC (Monmouth Junction, NJ, USA)) and the concentration was increased in a stepwise manner. After two months, the cells were able to grow in 1 µM gefitinib and were then continuously subcultured with 1 to 2 µM gefitinib for an additional 6 months, followed by single-cell cloning to establish gefitinib-resistant cell lines.

### 4.2. Cell Viability Assay

PC9-GR and HCC827-GR cells were seeded in 96-well plates at 5 × 10^3^ cells per well. After treatment with different drug concentrations, cell viability was evaluated with the CCK-8 reagent (Beyotime, Shanghai, China) at the indicated time points; the plates were incubated at 37 °C for 2 h, and absorbance was read at 450 nm on a BIO-TEK microplate reader (Winstock, VT, USA). IC_50_ values were calculated with GraphPad Prism 10.0 (San Diego, CA, USA).

### 4.3. Colony Formation Assay

Clonogenic assays were performed to evaluate the long-term anti-tumor efficacy of co-treatment with gefitinib and CDDO-Me (CAS 218600-53-4, 99.66% purity, #HY-13324, MedChemExpress, Shanghai, China) in NSCLC cells. PC9-GR and HCC827-GR cells were plated in 6-well plates (1 × 10^3^ cells per well), allowed to attach overnight, and then exposed to the indicated concentrations of CDDO-Me (0.6 µM), gefitinib (10 µM) and their combination. After 24 h, the drugs were removed and replaced with fresh complete medium; cultures were maintained for 10–14 days to permit colony formation. Colonies were subsequently rinsed with ice-cold PBS (#G4202-500ML, Servicebio, Wuhan, China), fixed with 4% paraformaldehyde for 15 min, and stained with 0.5% crystal violet at room temperature for 15 min. After thorough washing, colonies were counted macroscopically with an EVOS XL Core Imaging System (Thermo Fisher Scientific, Waltham, MA, USA).

### 4.4. Western Blot Analysis

Whole-cell lysates were prepared as described previously. Briefly, cells were incubated on ice for 10 min in RIPA lysis buffer (Beyotime, Shanghai, China) supplemented with complete protease inhibitors and cleared by centrifugation at 4 °C. Protein concentrations were determined with a bicinchoninic acid (BCA) protein assay kit (Beyotime, Shanghai, China). Equal amounts of protein were mixed with loading buffer, denatured at 95 °C for 5 min, resolved on 10–15% SDS-PAGE gels, and transferred to polyvinylidene fluoride (PVDF) membranes (Millipore, Billerica, MA, USA). After blocking with 5% non-fat milk for 1 h, membranes were probed overnight at 4 °C with the indicated primary antibodies diluted in TBST. Following three TBST washes, membranes were incubated for 1 h at room temperature with HRP-conjugated secondary antibodies, then washed three additional times, and developed with ECL reagent. The antibodies used were phospho-Src (Y419) (p-Src) (HUABIO, Hangzhou, Zhejiang, China, #ET1609-15, 1:1000), phospho-STAT3 (p-STAT3) (Y705) (HUABIO, Hangzhou, Zhejiang, China, #ET1603-40, 1:1000), STAT3 (HUABIO, Hangzhou, Zhejiang, China, #ET1605-45, 1:2000), Cytochrome-C (Cyto-C) (HUABIO, Hangzhou, Zhejiang, China, #ET1610-60, 1:2000), Src (HUABIO, Hangzhou, Zhejiang, China, #ET1702-03, 1:1000), PARP1 (HUABIO, Hangzhou, Zhejiang, China, #ET1608-56, 1:2000), Caspase-3 (HUABIO, Hangzhou, Zhejiang, China, #ET1602-39, 1:1000), Bcl-2 (D55G8) (CST, Danvers, MA, USA, #4223, 1:1000), Beta Actin (β-actin) (HUABIO, Hangzhou, Zhejiang, China, #EM21002, 1:10,000), Cleaved Caspase-3 (Cl-Caspase 3) (Asp175) (CST, Danvers, MA, USA, #9661, 1:1000), Bax (D2E11) (CST, Danvers, MA, USA, #5023, 1:1000), Cleaved PARP (Cl-PARP) (Asp214) (D64E10) (CST, Danvers, MA, USA, #5625, 1:1000), DFNA5/GSDME (HUABIO, Hangzhou, Zhejiang, China, #HA723251, 1:2000). The secondary antibodies uesd in this study were anti-rabbit IgG Fab2 (Sigma, St. Louis, MO, USA, #A0545, 1:10,000) and anti-mouse IgG Fab2 (Sigma, St. Louis, MO, USA, #A4416, 1:10,000). Signals were captured on a Bio-Rad ChemiDoc XRS system (Bio-Rad Laboratories, Hercules, CA, USA), and band intensities were quantified with ImageJ software (version 6.1).

### 4.5. Apoptosis Assay

To evaluate the effect of combined drug exposure on apoptosis, NSCLC cell lines were plated at 2 × 10^5^ cells per well in 6-well plates and treated for 24 h with CDDO-Me (0.6 µM), gefitinib (10 µM) and their combination; DMSO served as the vehicle control. Apoptotic fractions were quantified by Annexin V-FITC/PI staining using a Beckman Coulter CytoFLEX flow cytometer (Brea, CA, USA). Apoptosis plots were processed with FlowJo software (version 10.8.1).

### 4.6. Wound-Healing Assay

To evaluate the effect of drug treatment on cell migratory ability, PC9-GR and HCC827-GR cells were grown to 90–100% confluence in 6-well plates. A uniform wound was generated by scratching the monolayer with a sterile 10 µL pipette tip, after which detached cells were gently washed away with PBS. Cultures were then exposed to CDDO-Me (0.6 µM), gefitinib (10 µM) and their combination in RPMI-1640 supplemented with 1% FBS. Wound closure was documented at 0 and 24 h under a light microscope (200× magnification). The residual gap area was quantified with ImageJ, and migration rates were plotted as bar graphs using GraphPad Prism 10.0.

### 4.7. Transfection of siRNAs

PC9-GR and HCC827-GR cells were transfected with siRNA oligonucleotides using siRNA-mate plus Transfection Reagent (GenePharma Co., Ltd., Shanghai, China). The siRNAs were synthesized by GenePharma Co., Ltd. (Shanghai, China), and cells were used for subsequent experiments 24 h after transfection. The siRNA sequences targeting Src were as follows: siSrc #1, 5′-GGCUCAUUGAAGACAAUGATT-3′ and 5′-UCAUUGUCUUCAAUGAGCCTT-3′; siSrc #2, 5′-CUCUAUGACUAUGAGUCUATT-3′ and 5′-UAGACUCAUAGUCAUAGAGTT-3′.

### 4.8. Lactate Dehydrogenase (LDH) Release Assay

The cytotoxicity of gefitinib, CDDO-Me and their combination was analyzed by LDH release assay using an LDH Cytotoxicity Assay Kit (Beyotime, Shanghai, China). PC9-GR and HCC827-GR were seeded in a 96-well plate at a density of 1 × 10^4^ cells per well and treated with CDDO-Me (0.6 µM), gefitinib (10 µM) and their combination. Following treatment, the cells were centrifuged, and 120 µL of the supernatant was transferred to a new 96-well plate. To each well, 60 µL of LDH working solution (containing lactic acid, INT solution, and enzyme solution) was added and mixed thoroughly. After the 96-well plate was protected from light for 30 min, absorbance was detected at 490 nm using the microplate reader (BIO-TEK, Inc., Winooski, VT, USA). The results were analyzed with GraphPad Prism 10.0 software.

### 4.9. Reactive Oxygen Species (ROS) Measurement

ROS were quantified with the oxidant-sensitive probe 2′,7′-dichlorodihydrofluorescein diacetate (DCFH-DA). The ROS assay kit was purchased from Beyotime (Shanghai, China). PC9-GR and HCC827-GR cells were plated at 1 × 10^5^ cells per well in 6-well plates and exposed for 24 h to CDDO-Me (0.6 µM), gefitinib (10 µM) and their combination; DMSO served as the vehicle control. After treatment, the cells were harvested, centrifuged, and incubated with 10 µM DCFH-DA at 37 °C for 30 min in the dark. Cells were rinsed twice with PBS and immediately analyzed on a Beckman Coulter CytoFLEX flow cytometer (Brea, CA, USA). Mean fluorescence intensity was calculated with FlowJo v10.8.1 software.

### 4.10. Enhanced Mitochondrial Membrane Potential Assay Kit with JC-1

Mitochondrial membrane potential (MMP) was detected using the JC-1 Enhanced Mitochondrial Membrane Potential Detection Kit (Beyotime, Shanghai, China). PC9-GR and HCC827-GR cells were seeded into 6-well plates at a density of 1 × 10^5^ cells per well and exposed for 24 h to CDDO-Me (0.6 µM), gefitinib (10 µM) and their combination; DMSO served as the vehicle control. The cells were washed once with PBS and incubated with JC-1 working solution at 37 °C in a 5% CO_2_ incubator for 20 min. After incubation, the cells were washed twice with JC-1 assay buffer, centrifuged, and resuspended in JC-1 assay buffer. The treated cells were analyzed using a Beckman Coulter CytoFLEX flow cytometer (Brea, CA, USA). All analyses were performed with FlowJo v10.8.1 software.

### 4.11. Animal Experiments

Male BALB/c nude mice (5–6 weeks) were purchased from Jiesijie Experimental Animal Co. Ltd. (Shanghai, China). All mice were maintained under SPF conditions at a constant temperature (25 °C) and relative humidity (65%) with a 12 h light/dark cycle. PC9-GR cells (1 × 10^6^) were inoculated subcutaneously into mice 6–7 weeks of age. Tumor volumes were evaluated with calipers and calculated using the standard formula: volume = width^2^ × length × 0.5. Once the tumor volume reached approximately 100 mm^3^, mice were randomly separated into four groups (five mice per treatment group) and received intraperitoneal injections of CDDO-Me (7 mg/kg), gefitinib (10 mg/kg) and the combination of them, and the control group was injected with DMSO once every two days for 21 days, respectively. Tumor volume and body weight were monitored every 2 days. The protocols for animal care and euthanasia were approved by the Institutional Animal Care and Use Committee of Shanghai Ocean University (Shanghai, China) (Permit #SHOU-DW-2025-041).

### 4.12. Hematoxylin-Eosin (HE) and Immunohistochemical Staining

HE and Immunohistochemical staining were performed by Shanghai RecordBio Co., Ltd. (Shanghai, China). Tumor sections were immunostained with antibodies against phospho-Src, GSDME, and Ki-67. The images were captured using a Wisleap WS-10 scanner and analyzed with NDP.view 2.3.

### 4.13. Network Pharmacology Analysis

The drug targets of CDDO-Me and gefitinib were screened using the PubMed and SwissTargetPrediction databases. Subsequently, NSCLC-related genes were identified using the OMIM (https://www.omim.org/ (accessed on 18 September 2025)) and GeneCards (https://www.genecards.org/ (accessed on 18 September 2025)) databases, and the intersections were obtained using Venny 2.1.0 (https://bioinfogp.cnb.csic.es/tools/venny/ (accessed on 18 September 2025)). The results were analyzed using STRING (https://cn.string-db.org/ (accessed on 18 September 2025)) to obtain the protein-protein interaction network, and the exported TSV files were imported into Cytoscape 3.10.3 for network visualization and analysis.

### 4.14. Molecular Docking

Molecular docking was performed by AutoDock v.1.2.4 and AutoDock Vina v.1.2.0. The crystal structure of human c-Src (PDB ID: 1FMK) was retrieved from RCSB Protein Data Bank (http://www.pdb.org (accessed on 18 September 2025)). The 3D structure of CDDO-Me was generated using Chem3D v.19.0 and optimized using the MM2 force field for energy minimization. All missing terminal residues of the protein structure were repaired using Swiss-PdbViewer v.4.10. The protein was then prepared by removing crystallographic water, adding polar hydrogens, and assigning Kollman charges for docking studies.

Based on the ATP-binding site residues (276–284 and 298) and the active site residue (389) identified from UniProt and PROSITE annotations, the grid box was centered at coordinates x = −11.941, y = 19.433, z = 27.329. The grid dimensions were set to encompass the binding pocket defined by the coordinates: x: −20.6 to 10.1, y: −3.3 to 28.8, z: 21.1 to 33.6. All other docking parameters were kept at default settings. The protein–ligand binding interactions were analyzed and visualized using UCSF Chimera v.1.16 and BIOVIA Discovery Studio Visualizer v.21.1.0.20298.

### 4.15. Cellular Thermal Shift Assay (CETSA)

To validate the interaction between CDDO-Me and Src, a cellular thermal shift assay (CETSA) was performed in PC9-GR cells. PC9-GR cells were seeded in 6-well plates at a density of 2 × 10^5^ cells per well and cultured in complete medium. The cells were co-incubated with DMSO and CDDO-Me (2 μM) for 1 h, after which total protein was extracted using RIPA lysis buffer. The protein lysates were aliquoted into six PCR tubes and heated individually at the indicated temperatures ranging from 42 to 57 °C for 10 min. Finally, the samples were analyzed by Western blotting.

### 4.16. Data Analysis (GO and KEGG)

GO and KEGG enrichment analysis were conducted using the DAVID database (https://david.ncifcrf.gov/ (accessed on 23 September 2025)) and visualized with the Microbiology Letter online tool (https://bioinformatics.com.cn/ (accessed on 23 September 2025)).

### 4.17. Statistical Analyses

All experimental data were analyzed with GraphPad Prism 10.0 (GraphPad Software Inc, San Diego, CA, USA). Data are presented as mean ± SD from three independent experiments. For grouped data, statistical significance was determined using two-way ANOVA followed by Tukey’s post hoc test (* *p* < 0.05, ** *p* < 0.01, *** *p <* 0.001, **** *p* < 0.0001 vs. control or as indicated).

## 5. Conclusions

In summary, our study demonstrates that the combination of CDDO-Me and gefitinib can restore gefitinib sensitivity in EGFR-TKI-resistant NSCLC cells. Mechanistically, the combination suppresses the Src/STAT3 survival axis and induces mitochondrial stress while engaging pyroptosis through the Caspase-3/GSDME pathway. This concept provides a rationale for further evaluation of the combination strategy and for developing related compounds as sensitizing agents to be paired to overcome EGFR-TKI resistance in NSCLC.

## Data Availability

The original contributions presented in this study are included in the article/Supplementary Materials. Further inquiries can be directed to the corresponding author.

## Supplementary Materials

The following supporting information can be downloaded at https://www.mdpi.com/article/10.3390/ijms27146481/s1.
