# Supplementary figures and images for: CDDO-Me Overcomes Gefitinib Resistance in NSCLC by Targeting the Src/STAT3 Axis to Induce Apoptosis and Pyroptosis
Source: Int J Mol Sci. 2026 Jul 21;27(14):6481. doi: 10.3390/ijms27146481 (PMC13410134; doi:10.3390/ijms27146481)

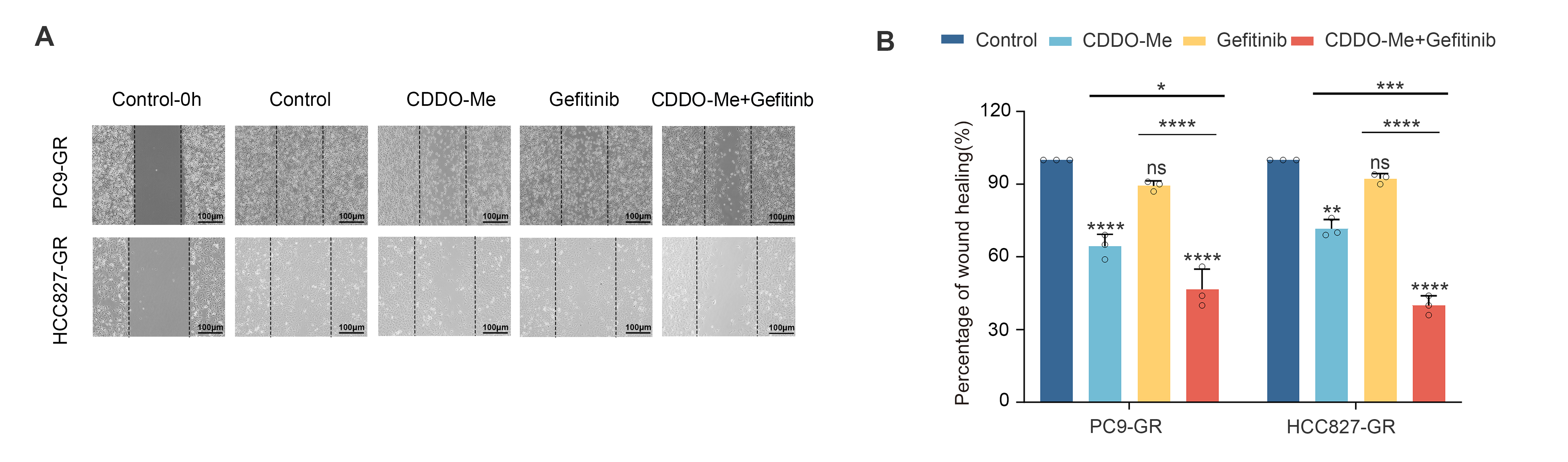

Supplement: Supplementary file 1 [file ijms-27-06481-s001.zip › Figure S1.tif]

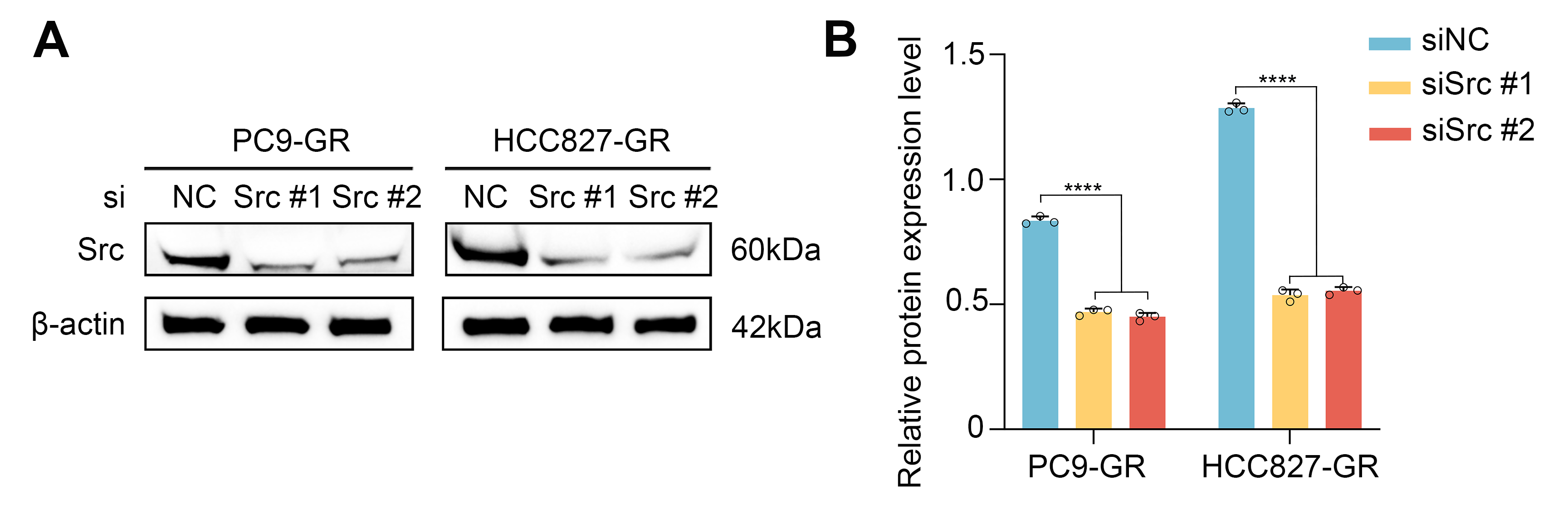

Supplement: Supplementary file 1 [file ijms-27-06481-s001.zip › Figure S2.tif]
